# Fundamentals of Arthroscopic Surgery Training and beyond: a reinforcement learning exploration and benchmark

**DOI:** 10.1007/s11548-024-03116-z

**Published:** 2024-04-29

**Authors:** Ivan Ovinnikov, Ami Beuret, Flavia Cavaliere, Joachim M. Buhmann

**Affiliations:** Department of Computer Science, ETH Zürich, Zurich, Switzerland

**Keywords:** Computer-assisted intervention, Reinforcement Learning, Benchmark, Surgical evaluation, Simulation

## Abstract

**Purpose:**

This work presents FASTRL, a benchmark set of instrument manipulation tasks adapted to the domain of reinforcement learning and used in simulated surgical training. This benchmark enables and supports the design and training of human-centric reinforcement learning agents which assist and evaluate human trainees in surgical practice.

**Methods:**

Simulation tasks from the Fundamentals of Arthroscopic Surgery Training (FAST) program are adapted to the reinforcement learning setting for the purpose of training virtual agents that are capable of providing assistance and scoring to the surgical trainees. A skill performance assessment protocol is presented based on the trained virtual agents.

**Results:**

The proposed benchmark suite presents an API for training reinforcement learning agents in the context of arthroscopic skill training. The evaluation scheme based on both heuristic and learned reward functions robustly recovers the ground truth ranking on a diverse test set of human trajectories.

**Conclusion:**

The presented benchmark enables the exploration of a novel reinforcement learning-based approach to skill performance assessment and in-procedure assistance for simulated surgical training scenarios. The evaluation protocol based on the learned reward model demonstrates potential for evaluating the performance of surgical trainees in simulation.

**Supplementary Information:**

The online version contains supplementary material available at 10.1007/s11548-024-03116-z.

## Introduction

Training procedures of surgical residents are heavily reliant on accumulated operative time as a metric of proficiency. The limited availability of in-vivo training opportunities, i.e. patient cases, can result in a suboptimal or even deficient training process of surgical novices [1] with far-reaching consequences.

Surgical simulators provide training scenarios which allow users to alleviate the bottleneck of low patient case numbers, but often use rudimentary evaluation metrics such as total procedure time and instrument path length.

Intra-operative performance assessment [2] plays a central role in answering the question how to evaluate a sequence of decision steps taken by a surgeon. Furthermore, it also provides valuable feedback how to improve on a given surgical task both for trainees and for experienced practitioners in continuing education.

In order to adapt the training process accordingly, we propose to model the behaviour of surgical trainees on simulator hardware in a principled way by considering a sequential decision-making agent interacting with a surgical environment. This interaction is formalised by the reinforcement learning (RL) framework. We envision a scenario where virtual agents trained using RL and inverse RL methods are utilised to provide real-time scoring and prediction of the trainees’ performance as well as to generate visual cues aimed at improving the performance or procedure guidance. Inverse RL (IRL) methods are particularly appealing due to their outstanding data efficiency and robustness to distribution shift [3] when compared to simpler imitation learning methods such as behavioural cloning (BC). Contrary to commonly studied surgical robotics settings employing RL [4, 5], this work primarily focuses on the study of training and evaluation signals characterised by a reward function that the agent receives on interaction with the simulated environment.

The simulated environment is realised as a set of surgical tasks derived from the Fundamentals of Arthroscopic Surgery Training (FAST) suite with the purpose of training dexterous manipulation of arthroscopic instruments by a surgical trainee. The FAST suite was designed in collaboration with various orthopaedic surgeon associations^1^ and is implemented in Unity3D by VirtaMed AG.^2^ It has been shown [6] that the training of fundamental skills involving dexterous manipulation of instruments using the FAST suite improves the performance of arthroscopic trainees in subsequent procedures. Hence, the FAST framework provides a surgically relevant skill basis for the purposes of simulated training. We demonstrate the utility of our algorithmic pipeline by evaluating a diverse set of human-recorded trajectories. The pipeline also suggests a selection of scoring functions for surgical performance.

This paper contributes the following scientific ideas and research solutions:

- A configurable interface for the FAST surgical training simulation (FASTRL).^3^ which allows easy deployment of standard RL and inverse RL algorithms. To our knowledge, this is the first benchmark aimed at improving both evaluation and assistance of surgical training in the context of RL, in contrast to robotic surgical environments.
- A new data-driven approach is based on expert demonstrations for evaluation of surgical skill performance using RL methods. We demonstrate the effectiveness of this evaluation on datasets recorded by experts and novice users of the simulation hardware.

## Related work

*RL frameworks for surgical robotics* Automation of surgical procedures holds promise of improving the surgical outcome and, hence, defines a long-standing goal in robotics for medicine. A number of benchmarks have previously been proposed which enable to perform the training of RL agents in a surgical robotics setting. In particular, the daVinci Research Kit (dVRK) [5] has been adopted as a popular platform by the surgical community. In [4], the use of RL is demonstrated for manipulation of soft tissues using a robot arm. Ho & Ermon employ an adversarial imitation method [8] in the context of surgical soft-tissue retraction using the dVRK platform [5]. Xu et al. propose an open-source platform simulator to replicate a series of manipulation tasks using the dVRK telerobotic platform that is specifically geared towards robotic learning. In contrast to previous work focused on training robotic policies, our benchmark design specifically suggests a scenario of surgical teaching assistance of human trainees. Furthermore, in our approach, we explicitly propose to use the reward function recovered via IRL methods for the purposes of trainee evaluation and guidance.

*Surgical skill assessment using machine learning methods* The use of machine learning for the analysis and performance of surgical procedures has previously been explored in robotic surgery [10–12], where both kinematic and visual data are readily available at procedure time. The demonstrated approaches typically apply various forms of supervised learning methods which exhibit a trade-off between how expressive models are and how many labelled examples they require for training. A number of methods ranging from score regression to deep neural network-based classification and segmentation of surgical procedures from videos have been presented in the literature [13, 14]. Typically, surgical skills are assessed post-operatively with data recorded during the procedure.

Contrary to established approaches, our system provides a dynamic real-time assessment of the surgical performance in simulated tasks as well as real-time feedback which can be queried by the trainee in the course of a procedure.

## Algorithmic pipeline for surgical assistance

**Fig. 1:**
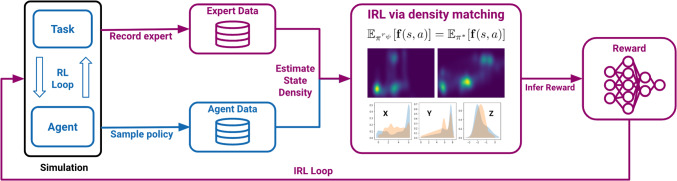
Algorithmic pipeline: a handcrafted reward heuristic allows the training of the agent, forming the *RL loop*. The recorded trajectories (synthetic or human) are then used as an input to the inverse RL algorithm which allows to recover a reward function based on state density matching between the expert and the learning agent (*IRL loop*). The resulting reward and policy are then utilised for training assistance

In this section, we motivate the design and development of the FASTRL set of tasks: (i) the algorithmic pipeline for surgical training assistance and (Fig. 1) (ii) the realisation of the FAST suite as an RL benchmark.

**Fig. 2:**
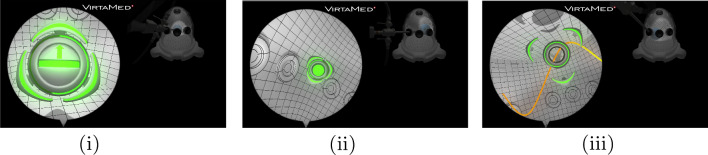
Benchmark environment tasks: (i) ImageCentring (ii) Periscoping (iii) TraceLines. The avatar highlighted in green displays visual cues to guide the training agents. The cues are encoded as part of the heuristic reward structure used for forward RL training

### Problem setting

*Simulated surgical training scenario* Arthroscopic surgical skills training in a computer simulation provides a demanding and scalable setup to investigate an educational training scenario for medical interventions. The trainee is challenged with a number of basic instrument handling tasks which are performed in an augmented reality (AR) simulator. The instruments, typically consisting of an arthroscope, a palpation hook and a grasper, are inserted into the half-dome structure through portals, and the output of the arthroscopic camera is simulated on a screen (Fig. 2) depicting the surgery environment. The simulated setting has a number of benefits, including potentially unlimited interaction capacity between the trainee and the surgical environment as well as access to a variety of data modalities for analysis purposes, that are often difficult to obtain in real-world settings with patients. We aim to enhance the simulated training scenario with evaluation and assistance based on the formalisation of the interaction between simulator and trainee as an RL problem.

*RL formalism* We model the simulation environment for surgical procedures as a *Markov decision process* (MDP) ${\mathcal {M}}= ({\mathcal {S}}, {\mathcal {A}}, {\mathcal {T}}, P_0, R)$, where ${\mathcal {S}}$ is the state space, ${\mathcal {A}}$ is the action space, ${\mathcal {T}}: {\mathcal {S}}\times {\mathcal {A}}\rightarrow {\mathcal {S}}$ is the transition function, $P_0$ is the initial state distribution, and $R:{\mathcal {S}}\times {\mathcal {A}}\rightarrow \mathbb R$ is the reward function. A *policy* $\pi : \mathcal {S} \rightarrow \mathbb {R}_+, s \mapsto \pi (a|s)$ is a conditional probability distribution of actions $a\in {\mathcal {A}}$ given states $s \in {\mathcal {S}}$ with state features $\varphi (s)$.

**Fig. 3:**
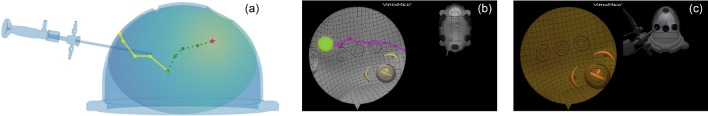
Assistant illustration. **a** path suggestion (green line) based on performed trajectory (yellow line) in the FAST dome with reward potential around target (star), **b** path suggestion from camera POV, **c** colour cue for the trainee’s current state

In the *inverse reinforcement learning* (IRL) setting, the reward is unknown and a suitable parametric reward function $r_\psi$ is estimated based on a dataset of *expert trajectories* $\mathcal {D}_E = \{\tau _i\}_{i\le K}$ where $\tau _i = (s_{1:T}^{(i)},a_{1:T}^{(i)})$ is a sequence of states and actions of expert *i* of length *T*. To achieve this goal, various distribution matching methods can be used [8, 15, 16]. Under the assumption of fixed transition dynamics $\mathcal {T}$, the reward function defines a succinct representation of the task to be performed and it elicits desired behaviours guided by RL algorithms [3]. In the context of a simulated environment, this learning control enables reward functions to serve as a central part of evaluation schemes for human trainees.

We consider two learning modalities in our pipeline:

- *Forward RL*: model-free RL methods based on reward *heuristics* designed by *human experts*.
- *Inverse RL*: inverse setting where the reward function is *directly inferred* from a set of expert demonstrations.

In the first modality, we define heuristic reward functions which enable training of virtual agents that learn to complete the benchmark tasks. The benchmark tasks require a level of sophistication which depends on the design of multiple reward components in order to elicit correct behaviours. This modality enables the training of agents which learn to mimic surgical behaviour via the definition of a suitable reward function and standard model-free RL method implementations. This modality mainly intends to explore the underlying search space of surgically correct procedures by specifying heuristic evaluation schemes. It can also enable experts to generate virtual agents by specifying a set of preference weights for the behavioural components of the heuristic reward function via the provided API.

In the inverse modality, an extrinsic reward function is not explicitly defined; instead, a reward function is inferred from the union set of the recorded expert demonstrations on the respective task. This modality directly allows users to be evaluated with respect to a *learned* reward function, as opposed to a hand-designed heuristic. The combination of these two modalities allows us to obtain a reward and value function tuple $(r_\psi , V_\phi )$ and the policy $\pi _{r_\psi }$ which has been trained to optimise $r_\psi$ derived from a set of expert demonstrations $\mathcal {D}_e$.

Both the policy and the reward function can be utilised as *feedback* mechanisms for human learners. These functions support real-time contextual evaluation of the trainee’s performance as well as a quantitative prediction of the procedure outcome, respectively.

In addition, the trainee can query the virtual agent policy at any point in the procedure to generate a demonstration of the next steps based on the current trainee state using the simulated dynamics. This assistance modality is illustrated in Fig. 3.

In order to explore this setting, we propose a standard RL benchmark set to train virtual agents aimed at surgical assistance of arthroscopic procedures. An example pipeline consisting of forward and inverse RL algorithms is illustrated in Fig. 1.

### Benchmark environment

We use the Arthros®FAST (Fundamentals of Arthroscopic Surgery Training) simulator implemented in Unity3D and provided by Virtamed AG to evaluate the RL pipeline. The simulation provides a number of educational navigation and manipulation tasks that have to be performed within a hollow dome structure (Sawbones®FAST workstation) in accordance with the FAST (Fundamentals of Arthroscopic Surgery Training) training problem.^4^ We consider three tasks for our benchmark environment: image centring and horizoning (ImageCentring), which trains basic endoscope manipulation and monocular depth estimation, periscoping (Periscoping), which trains the use of angled optics and line tracing (TraceLines), which trains steady instrument movement. The tasks consist of guiding the tip of the virtual arthroscope to various locations marked by an avatar that displays visual cues. The trainee or the virtual agent pursues the goal of orienting the arthroscope in such a way that it complies with the cues and that it centres the image of the avatar in the field of view of the endoscope camera.

*MDP formulation* In order to solve the tasks using the RL framework, we formalise it as the following continuous control problem: a state $s_t$ in the continuous *state space* $\mathcal {S}$ is defined as the concatenation of the three-dimensional position $\textbf{x}_t^{\text {arth}}$ and rotation quaternion $Q_t^{\text {arth}}$ of the arthroscope as well as the respective time derivatives $\dot{\textbf{x}}_t^{\text {arth}}$ action space and $\dot{Q}_t^{\text {arth}}$. Additionally, we provide the position and rotation of the target avatar $\textbf{x}_t^{\text {tgt}}, Q_t^{\text {tgt}}$ as well as the cross-product between the forward vector of the arthroscope camera and the forward vector of the avatar transform $\alpha _t^{\times (\text {arth},\text {tgt})}$, which describes the alignment between the arthroscope camera and the avatar. The full-state specification is given as the following tuple: $s_t = (\textbf{x}_t^{\text {arth}}, Q_t^{\text {arth}}, \textbf{x}_t^{\text {arth}}, \dot{\textbf{x}}_t^{\text {arth}}, \dot{Q}_t^{\text {arth}}, \textbf{x}_t^{\text {tgt}}, Q_t^{\text {tgt}}, \alpha _t^{\times (\text {arth},\text {tgt})}) \in \mathcal {S}$. We propose two versions of the action space. The seven-dimensional continuous *action space* $\mathcal {A}$ consists of stacked acceleration and angular acceleration vectors as well as the light cable rotation angle $\omega _c$ of the arthroscope, which realises an independent camera angle control $a_t = (\ddot{\textbf{x}}_t, \ddot{\mathbf {\phi }}_t, \omega _c) \in \mathcal {A}$. The alternative five-dimensional *discrete action space* $\mathcal {A}_d$ is defined as the translation of the arthroscope along its axis $x_t$, changes in pivoting angle around the portal $\Delta \alpha _p, \Delta \beta _p$, rotation around the translation axis $\Delta \delta _p$ and the rotation of the camera $\omega _c$. The *transition function* $f(s_t, a_t) \in \mathcal {T}$ is implemented by the Unity physics engine, which simulates the friction coefficients of the arthroscopic entry point. The physical interaction is deterministic for most use cases.

*Reward shaping* Eliciting successful behaviour crucially requires a correct specification of the reward. During the procedure, the simulation provides visual avatar cues to the agent which specifies a sparse reward scheme. In order to increase the sample efficiency of the training process, we additionally define a dense linear reward. The heuristic reward function $r_\text {heur} = \textbf{w}^T \phi (s_i)$ is defined as a weighted sum of the reward features $\phi (s_i)$.^5^ The reward features and corresponding reward weights are configurable at runtime and are exposed to the user. In particular, this allows the experimentalist to explore the generation of behaviours based on various combinations of reward features as well as multi-objective optimisation methods.

### Learning algorithms

The virtual agents are parameterised using an actor-critic architecture which consists of the policy function $\pi _\theta$ and either the value function $V_\phi (s)$ (on-policy) or state-action value function $Q_\phi (s,a)$ (off-policy). $V_\phi (s)$ approximates the discounted empirical returns $\hat{V}(s) = \sum _{t\le T} \gamma ^t r(s_t)$ obtained on rollouts of the policy. The agents are trained employing a variant of the *proximal policy gradient* (PPO) [17] algorithm. When learning from human trajectories, we utilise an off-policy algorithm, *soft actor-critic* (SAC) [18], for purposes of sample efficiency.

To solve the inverse RL problem with maximum entropy regularisation as outlined in Section “Problem setting”, we utilise the adversarial imitation learning approach [8].

Adversarial inverse RL (AIRL) methods yield a reward function by learning to distinguish between the transitions sampled from the dataset of expert trajectories $\tau \sim \mathcal {D}_E$ and transitions continually sampled from the rollout buffer of the improving policy $\tau \sim \mathcal {D}_\pi$. The policy $\pi _\theta$ maximises an expected cumulative reward objective based on the discriminator output. The discriminator is optimised using the binary cross-entropy loss. We utilise both the standard discriminator structure described in [8] and the shaped structure from [16] which separates into the state-dependent reward function $r_\psi$ and the state-dependent shaping term. The reward function specifically is used for downstream evaluation of novel trajectories. The *off-policy formulation* additionally necessitates two algorithmic additions. The first is spectral norm regularisation [19], a method which enforces the Lipschitz smoothness of the discriminator and stabilises the adversarial training procedure. The second is a modified sampling procedure for discriminator training, only utilising samples from the current policy rollout as opposed to mixture policy samples from the replay buffer. We demonstrate the impact of these additions in the experimental section.

## Experiments

The algorithmic pipeline for surgical assistance (Fig. 1) allows us to evaluate of human demonstrations using heuristic and learned rewards. In this section, we demonstrate its applicability on a test set of human trajectories gathered using the simulator. Furthermore, we measure the performance of various state-of-the-art methods in forward and inverse RL to illustrate the learning complexity of the benchmark tasks.

*Learning from demonstrations* In the first set of experiments, we demonstrate the ground truth task performance of the algorithms used to recover the reward and value functions on the proposed benchmark suite.

**Fig. 4:**
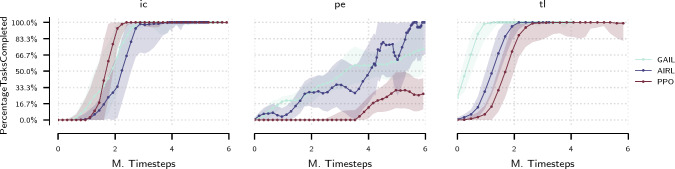
Comparison of forward and inverse RL experiments: median of percentage of tasks completed for ImageCentring (ic), Periscoping (pe) and TraceLines (tl) environments

We obtain the demonstration set by sampling trajectories from the best performing policy trained using the heuristic reward formulation described in Section “Benchmark environment”. In Fig. 4 we observe that inverse methods (GAIL, AIRL) converge to the desired solution faster for the Periscoping and TraceLines tasks than the forward RL method (PPO) but require more time to converge to the optimal solution in the ImageCentring case. This behaviour can be attributed to the degree of alignment between heuristic reward components and state space specification, which varies across tasks, making ImageCentring easier to solve in the forward learning scenario (PPO). Furthermore, the IRL method (AIRL) outperforms the pure imitation learning method (GAIL) in the Periscoping task. The discrepancy between GAIL and AIRL is ascribed to different divergence measures realized by the algorithms. Furthermore, the smaller number of parameters of the GAIL discriminator may explain faster initial convergence. Both policies trained using the forward RL method (PPO) and the ones obtained via inverse methods (GAIL, AIRL) can be used to generate trajectory suggestions in the assistance setting. The exact hyperparameters and experimental settings can be found in Appendix E.

**Fig. 5:**
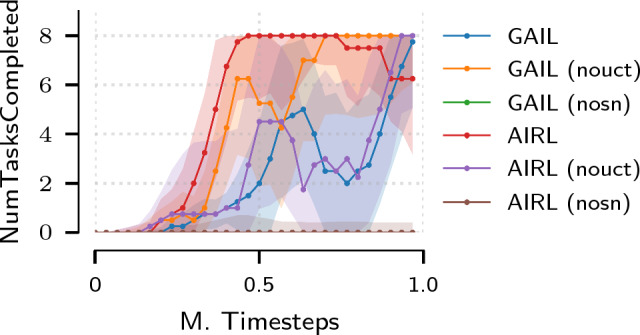
Ablation experiments for off-policy version of the presented algorithms: number of tasks completed for the TraceLines environment. The label nouct denotes the use of samples from entire replay buffer for specification of the density matching objective; nosn corresponds to the removal of spectral normalisation of the discriminator

In addition to evaluating the pipeline using synthetic demonstrations sampled from a policy trained on heuristic reward, we learn a policy and reward function from a set of human trajectories recorded using the keyboard and mouse user interface in the FASTRL API. We perform the evaluation on the TraceLines task. In Fig. 5 we can observe that, in order to attain sample efficient training and successfully complete all subtasks, the algorithmic modifications outlined in Section “Learning algorithms” are necessary.

**Table 1:** Trajectory scores (heuristic and learned rewards and values) for human (fm, an, io, mk, mv) and virtual (fw) agents on the Periscoping task. Here, $r_\psi$ denotes the learned reward, $V_\phi$ the learned value and $r_{heur}$ the reward heuristic

Agent ID	$r_\psi$	$V_\phi$	$r_{heur}$	Trajectory length
fw	$0.993_{\pm 0.004}$	$0.876_{\pm 0.076}$	$0.999_{\pm 0.0002}$	$1600.6_{\pm 971.0}$
fm	$0.732_{\pm 0.019}$	$0.728_{\pm 0.016}$	$0.742_{\pm 0.016}$	$1821.5_{\pm 104.8}$
an	$0.720_{\pm 0.042}$	$0.716_{\pm 0.040}$	$0.729_{\pm 0.039}$	$1929.2_{\pm 277.4}$
mk	$0.617_{\pm 0.117}$	$0.627_{\pm 0.121}$	$0.634_{\pm 0.106}$	$2578.5_{\pm 745.8}$
mv	$0.518_{\pm 0.094}$	$0.521_{\pm 0.094}$	$0.529_{\pm 0.095}$	$3313.5_{\pm 665.5}$
io	$0.374_{\pm 0.271}$	$0.339_{\pm 0.249}$	$0.384_{\pm 0.278}$	$4290.8_{\pm 1981}$

*Performance evaluation* In this section, we evaluate two sets of human trajectories using the reward and value functions obtained by both AIRL and GAIL algorithms as scoring functions. The first set of trajectories consists of three novices (io, mk and mv) and two expert practitioners fm and an. These trajectories have been recorded using the physical hardware platform.^6^ The second set of trajectories has been recorded using the keyboard-mouse interface and features three distinct skill levels defined based on the percentage of completed subtasks.

**Fig. 6:**
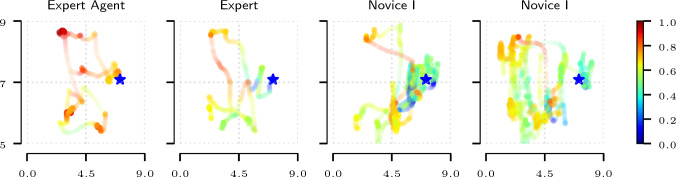
Trajectory projection (XY plane) comparison for Periscoping. Colours indicate the normalised value output by the learned evaluation function on the full states of the recorded trajectories. The star symbol indicates the start and end point of the trajectory. The alpha value encodes the time the agents spend in the evaluated states. *Expert Agent* outperforms human subjects. *Expert* human subject has a shorter and smoother trajectory compared to the novices which is reflected in the accumulated reward. The demonstrations were all recorded under the same conditions, from the same starting point. The end point might be different as different agents have different ways of solving the task

**Fig. 7:**
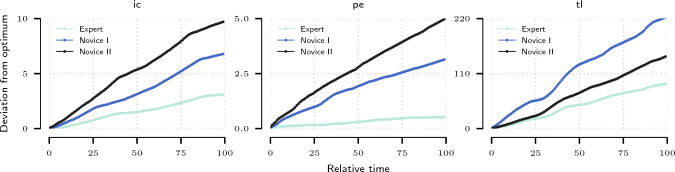
Comparison of deviation from the optimum by the expert and novice agents over the course of procedure. The error is integrated for every percent of procedure completed

In Table 1, we observe that both normalised value function and reward function recover the correct ranking among the five sets of trajectories. The learned reward and value functions are consistent with both the heuristic reward baseline and the trajectory length metric used in the simulator as the standard evaluation option. In Fig. 6, we plot the spatial *x* and *y* components of the states evaluated using the learned value functions. We observe a clear distinction between the novice and expert practitioners with the expert agent outperforming the human expert in terms of the score. In Fig. 7, we show a comparison of the agents from Fig. 6 in terms of the deviation from the expert agent reference over the course of the procedure. Again, a clear distinction between the skill levels of agents is observed. The distinction is consistent with respect to the ground truth level of expertise of the agents.

Table 2 summarises the results obtained on the reward learned on human trajectories. We observe a similar result in terms of the recovered ranking. The validation of learned reward functions remains an open question. Evaluation of synthetic policies typically relies on rollout performance on the ground truth reward, which is not directly applicable to the setting of human evaluation due to lack of a ground truth metric which would capture all relevant aspects of the behaviour.

As future work, validation by an independent expert using standardised evaluation protocols [20, 21] could be explored to assess the validity of the proposed evaluation metrics.

*Extension to laparoscopic setting* In this section, we provide a demonstration of the proposed method on a more challenging task, the diagnostic tour of the abdomen performed by surgeons in the context of laparoscopic surgery. The task goal consists of visualising a fixed sequence of anatomical landmarks in the abdominal area. The simulated version of this task is realised using a simplified version of the VirtaMed Laparos®^7^ hardware platform. The main goal of this experiment is to demonstrate the applicability of the algorithmic pipeline described in Fig. 1 to a more sophisticated surgical task, which features a larger cohort of demonstrations. In particular, this task features both realistic textures and significantly more complex anatomical constraints inherent to the abdominal area compared to the FAST setting. Due to the more challenging nature of the full diagnostic tour, we use a subset of the tour which focuses on visualizing both liver lobes and the falciform ligament. Furthermore, we restrict the state space of the MDP $\mathcal {S} \subseteq \mathbb {R}^3$ to correspond to the position of the endoscope camera.

**Table 2:** Normalised trajectory scores (heuristic and learned rewards and values) for human trajectories *Expert*, *Intermediate* and *Poor* on TraceLines task

Subject	$r_\psi$	$V_\phi$	$r_{heur}$	Trajectory length
*Expert*	$0.79_{\pm 0.20}$	$0.91_{\pm 0.05}$	$0.91_{\pm 0.035}$	$2928.2_{\pm 167.67}$
*Intermediate*	$0.30_{\pm 0.03}$	$0.64_{\pm 0.02}$	$0.55_{\pm 0.022}$	$2126.0_{\pm 90.48}$
*Poor*	$0.17_{\pm 0.035}$	$0.33_{\pm 0.035}$	$0.23_{\pm 0.027}$	$1111.2_{\pm 122.37}$

We evaluate the scoring functions obtained using the proposed method on a selection of human trajectories recorded as part of a surgical proficiency course.^8^ We train our method using 10 trajectories, which were rated best using the internal heuristic score provided by the simulator. We use two *scoring* metrics: (i) *the economy score:* the total distance covered by the endoscope camera within the abdominal cavity and (ii) *the safety score:* the sum of the Euclidean distances between the endoscope camera position and the closest anatomy vertices.

**Fig. 8:**
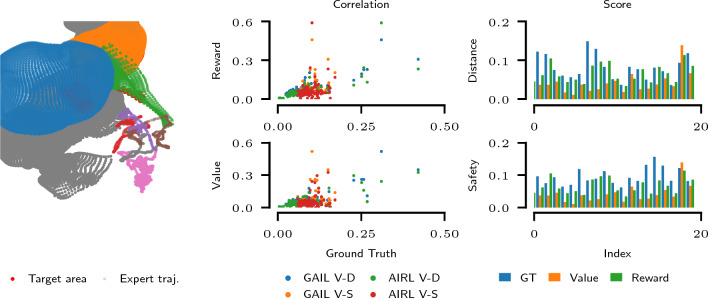
Laparoscopic trajectory evaluation. The left figure depicts the part of the anatomy to be visualized (liver lobes and falciform ligament) and corresponding trajectories. The plots on the right illustrate the trajectory scores and correlations w.r.t. ground truth metrics (V-D: Value-Distance, V-S: Value-Safety)

We evaluate a total of 100 trajectories using the recovered reward and value functions from both GAIL and AIRL formulations and summarize the results in Fig. 8 and Table 3. We can observe a strong correlation in terms of the Spearman rank correlation coefficient between both the recovered reward function and the scoring metrics as well as the recovered value function and the scoring metrics. The total instrument path length metric is strongly correlated with both the learned reward and the learned value. The safety metric exhibits a weaker correlation which is still significant according to the observed p-values.

**Table 3:** Correlation coefficients (p-values in brackets) between laparoscopic trajectories evaluated using recovered rewards and two ground truth metrics (*Dst*: total instrument distance and *Sft*: total safety distance)

Model	Reward-Dst. (p)	Reward-Sft. (p)	Value-Dst. (p)	Value-Sft. (p)
GAIL	$0.73\pm 0.03$ (0.00)	0.25 ± 0.02 (0.01)	$0.78\pm 0.01$ (0.00)	0.24 ± 0.01 (0.02)
AIRL	$-$ $0.82\pm 0.01$ (0.00)	$-$ $0.25 \pm 0.01$ (0.01)	0.80 ± 0.00 (0.00)	0.24 ± 0.00 (0.02)

## Discussion

In this paper, we have presented FASTRL, an RL benchmark for training virtual assistant agents in the arthroscopic surgery domain. We have investigated the performance of a number of state-of-the-art forward and inverse RL approaches and illustrated a prototypical solution to a surgical evaluation and assistance framework for arthroscopic procedures using model-free RL techniques. We have performed a feasibility study using our API extension of the simulation software provided by VirtaMed AG.

*Limitations* At the current state, only three tasks have been converted and evaluated. More sophisticated scenarios involving additional instruments such as the palpation hook or the grasper are in development and will be provided at a later stage. Currently, the pipeline has solely been evaluated on kinematic data, but the possibility exists to train image-based models on the rendered arthroscope camera images.

### Supplementary Information

Below is the link to the electronic supplementary material.

Supplementary file 1 (pdf 3877 KB)

## Footnotes

1 ABOS, AAOS, AANA.

2 virtamed.com, the environments are provided in binary format and accessible via the FASTRL API.

3 Fundamental of Arthroscopic Surgery Training using Reinforcement Learning (FASTRL). The API and the executables for the presented environments are made public and are available under: fastrl.ethz.ch The executables are distributed under the CC BY-NC-ND license. Video material and further details are available under the same URL.

4 Developed by American orthopaedic associations ABOS (American Board of Orthopaedic Surgery), AAOS (American Academy of Orthopaedic Surgeons) and AANA (Arthroscopy Association of North America.)

5 A detailed overview of the features is provided in Appendix.

6 We defer the details of data gathering procedure to appendix.

7 https://www.virtamed.com/en/products-and-solutions/simulators/laparos.

8 Details are provided in Appendix C.
