# Supplementary material for: Fundamentals of Arthroscopic Surgery Training and beyond: a reinforcement learning exploration and benchmark
Source: Int J Comput Assist Radiol Surg. 2024 Apr 29;19(9):1773–81. doi: 10.1007/s11548-024-03116-z (PMC11365860; doi:10.1007/s11548-024-03116-z)
Supplement: Supplementary file 1 — (pdf 3877 KB) [file 11548_2024_3116_MOESM1_ESM.pdf]

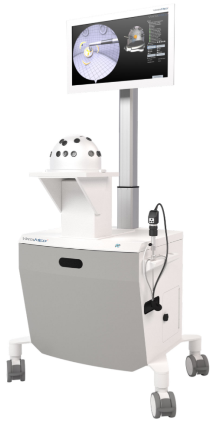

Fig. 9: VirtaMed FAST

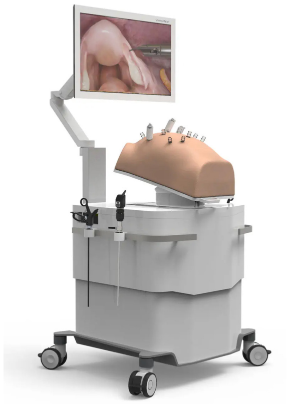

Fig. 10: VirtaMed Laparos

A Hardware platform

The hardware platform that serves as basis for the RL benchmark is the VirtaMed FAST simulator as shown in fig. 9. The simulator consists of a hollow dome structure with instrument entry portals for a selection of arthroscopic surgical tools (scope, hook, grasper). The output of the virtual arthroscopic camera as well as an optional third-person view of the dome structure are simulated on screen. The evaluation dataset for the performance ranking experiments (section 4) has been recorded using the hardware platform. Figure 10 depicts Laparos, the simulator platform adapted for laparoscopic surgery, which was used to record the expert and user demonstrations in section 4.

A.1 Computational resources

The experiments were executed on a GPU server equipped with Intel Xeon Gold 6150 and AMD EPYC 7742 CPUs and NVIDIA GTX1080Ti GPUs. The average running time per experiment are shown below. For the experiments in section 4, we use an extension of the Unity `ml-agents` framework as algorithmic implementation.

Algorithm	`ImageCentering`	`Periscoping`	`TraceLines`
PPO	143	151	149
SAC	186	197	194
GAIL (PPO)	202	229	226
AIRL (PPO)	180	192	182
GAIL (SAC)	285	321	304
AIRL (SAC)	242	239	243

Table 4: Overview of the average algorithm runtimes (in minutes per 1M environment interactions) for the different benchmark environments.

B API description

We provide an interface to algorithm implementations of two popular frameworks: an extension of the `ml-agents` [21] framework for the PPO with curiosity exploration algorithm and the inverse RL algorithms as well as `stable-baselines3` [22] for a more broad selection of baseline forward RL algorithms. Furthermore, the simulation parameters such as heuristic reward weights, target positions and curricula are exposed to the user as well. For the full documentation, we refer the reader the project website: `fastrl.ethz.ch`.

C Dataset details

FAST Dataset. For the purpose of skill performance evaluation, a dataset was obtained by subjects performing the three benchmark tasks using the simulator platform. Five subjects with different levels of expertise were invited to perform each of the three benchmark tasks for a total number of 5 repetitions. The trajectories were recorded according to the state space specification described in section 3.2. Two subjects were already very familiar with all tasks and are considered as *Experts*, while others with little or no experience with the simulation are considered as *Novices* as shown in table 5. Additionally, the participants are graded based on the length of their performed trajectories. This metric coincides with the default heuristic used by the VirtaMed simulator platform. For the evaluation performed in section 4, the subjects used the physical hardware described in appendix A, recorded using a magnetic sensor, which tracks instrument movement. In the second evaluation, the keyboard-and-mouse interface was used to record the trajectories.

Table 5: Participant performance ranking

Subject ID	Expertise	Trajectory length
fm	*Expert*	$1600.6_{\pm 971.0}$
an	*Expert*	$1929.2_{\pm 277.4}$
mk	*Novice*	$2578.5_{\pm 745.8}$
mv	*Novice*	$3313.5_{\pm 665.5}$
io	*Novice*	$4290.8_{\pm 1981}$

Laparoscopic diagnostic tour dataset. The diagnostic tour dataset used in section 4 was gathered using the laparoscopy simulator, where users were asked to perform a guided diagnostic tour of the abdomen with a fixed sequence of anatomical landmarks highlighted on screen. A total of 100 trajectories from a diverse set of experienced practitioners were obtained. The average reported proficiency based on the simulator evaluation (max. attainable score 150) was 128.24 ± 18.32 points.

D Additional results

This section provides additional results obtained using the algorithmic pipeline in fig. 1 and partially described in section 4.

D.1 Ablation study on reward components

In this set of experiments, we demonstrate the dependence of the forward RL methods on the reward shaping scheme. Due to the intricate task structure which includes a combination of multiple optimisation objectives, we introduce a number of additional reward components in order to improve the sample efficiency of the algorithms solving the tasks. We perform an ablation study on the reward components as shown in fig. 11 for the three tasks in order to demonstrate the necessity of reward shaping. The evaluation is carried out using the on-policy version of the policy algorithm (PPO). The five ablation settings comprise the baseline reward structure (`baseline`), the removal of distance and angle potentials respectively (`noDist`, `noAngle`, `noDistNoAngle`) and the sparse reward setting where a reward is only given if the target is visualised correctly (`onlyTaskCompleted`). The plots report the median of tasks completed over 5 randomly seeded repetition experiments per ablation setting. We can observe that potential based reward shaping on both Cartesian and angular components is crucial to obtain a policy which solves all targets successfully. We can observe a similar behaviour on the `Periscoping` task. In the case of `TraceLines`, the gradation is less distinct due to the specifics of the task. The visual components of the reward are statistically more prevalent in the `TraceLines` task and allow for a more dense reward structure also in absence of the distance and angle penalties. The sparse reward signal (`onlyTaskCompleted`) fails to solve a single target.

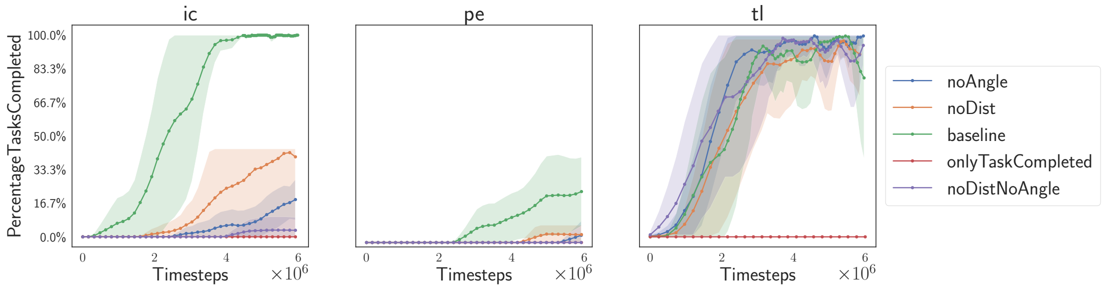

Fig. 11: Ablation experiments: number of tasks completed for `ImageCentering` (ic), `Periscoping` (pe) and `TraceLines` (tl) environments.

Table 6: Trajectory scores (rewards and values normalised) for human and virtual agents on the `ImageCentering` task

Agent ID	r_ψ	V_ϕ	r_{heur}	Trajectory length
fw	$0.998_{\pm 0.002}$	$0.680_{\pm 0.104}$	$0.999_{\pm 0.0002}$	$966_{\pm 782.0}$
fm	$0.610_{\pm 0.034}$	$0.773_{\pm 0.180}$	$0.742_{\pm 0.016}$	$1180.5_{\pm 104.9}$
an	$0.654_{\pm 0.046}$	$0.408_{\pm 0.213}$	$0.729_{\pm 0.039}$	$1053.5_{\pm 130.3}$
io	$0.462_{\pm 0.302}$	$0.499_{\pm 0.254}$	$0.384_{\pm 0.278}$	$1646.5_{\pm 925.3}$
mk	$0.611_{\pm 0.079}$	$0.543_{\pm 0.235}$	$0.634_{\pm 0.106}$	$1140_{\pm 197.9}$
mv	$0.684_{\pm 0.008}$	$0.402_{\pm 0.083}$	$0.529_{\pm 0.095}$	$956_{\pm 13.45}$

Table 7: Trajectory scores (rewards and values normalised) for human and virtual agents on the `TraceLines` task

Agent ID	r_ψ	V_ϕ	r_{heur}	Trajectory length
fw	$0.985_{\pm 0.007}$	$0.975_{\pm 0.015}$	$0.999_{\pm 0.0002}$	$4548.8_{\pm 881.5}$
fm	$0.668_{\pm 0.006}$	$0.630_{\pm 0.045}$	$0.677_{\pm 0.006}$	$3705.6_{\pm 45.5}$
an	$0.495_{\pm 0.072}$	$0.281_{\pm 0.039}$	$0.476_{\pm 0.065}$	$2295.3_{\pm 450.2}$
io	$0.399_{\pm 0.269}$	$0.293_{\pm 0.232}$	$0.389_{\pm 0.266}$	$4310.8_{\pm 1854.6}$
mk	$0.518_{\pm 0.159}$	$0.544_{\pm 0.051}$	$0.542_{\pm 0.071}$	$3237.6_{\pm 506.27}$
mv	$0.363_{\pm 0.067}$	$0.439_{\pm 0.119}$	$0.407_{\pm 0.037}$	$4224.0_{\pm 255.2}$

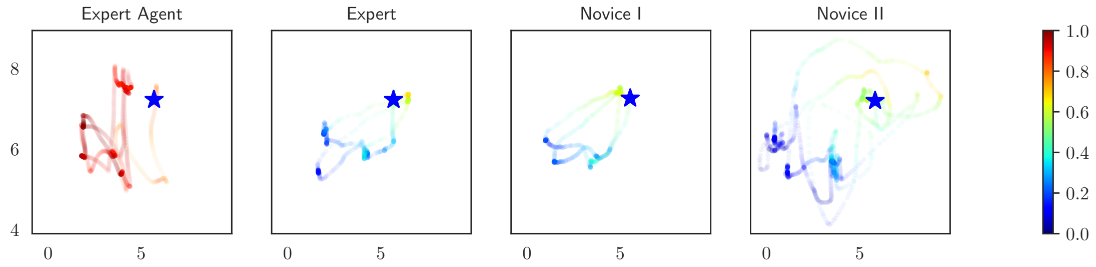

Fig. 12: Trajectory projection comparison for `ImageCentering`.

E Algorithmic details and SB3

E.1 `stable-baselines3` Results

We have evaluated a number of standard RL algorithm implementations of the `stable-baselines3` on our benchmark. Due to a high variance and a lack of tuned hyperparameters, we report the number of tasks completed by the best model from a set trained over 5 random seeds for every algorithm using a standard set of hyperparameters provided by the `stable-baselines3` framework.

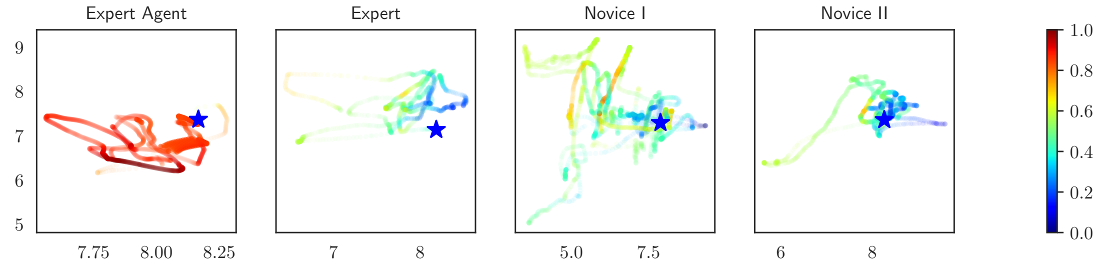

Fig. 13: Trajectory projection comparison for `TraceLines`.

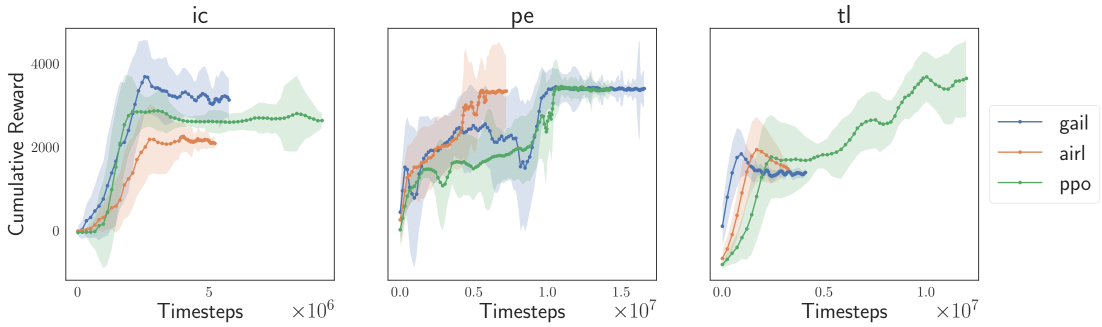

Fig. 14: Comparison of forward and inverse RL experiments: Cumulative reward for `ImageCentering` (ic), `Periscoping` (pe) and `TraceLines` (tl) environments.

Table 8: Normalised scores (heuristic and learned rewards and values) for human trajectories *Expert*, *Intermediate* and *Poor* on `ImageCentering` task

Subject	r_ψ	V_ϕ	r_{heur}	Traj. length
Expert	$0.98_{\pm 0.06}$	$0.93_{\pm 0.04}$	$0.95_{\pm 0.04}$	$1475.33_{\pm 69.16}$
Intermediate	$0.81_{\pm 0.06}$	$0.76_{\pm 0.06}$	$0.34_{\pm 0.026}$	$1279.0_{\pm 128.88}$
Poor	$0.40_{\pm 0.13}$	$0.41_{\pm 0.11}$	$0.13_{\pm 0.037}$	$720.4_{\pm 192.86}$

E.2 Algorithm hyperparameters (`ml-agents`)

This section provides an overview of the hyperparameters used to train the algorithms described in section 4.

Algorithm	ImageCentering	Periscoping	TraceLines
PPO	**6/6**	4/5	**8/8**
SAC	**6/6**	**5/5**	**8/8**
TQC	**6/6**	**5/5**	4/8
ARS	1/6	None	2/8
TD3	**6/6**	3/5	**8/8**
DDPG	None	None	None
TRPO	None	None	None

Table 9: Number of tasks completed using `stable-baselines3` algorithm implementations

E.3 Heuristic reward structure

The reward structure used for training in the forward modality uses a scalarisation approach based on a number of objectives. We present an overview of the individual objective terms in table 12. The objectives consist of dense reward potentials in positional and rotational spaces (*distance penalty* and *angle penalty* in table 12) as well a number of binary reward components such as various visualisation targets which vary across tasks.

F Expert trajectories

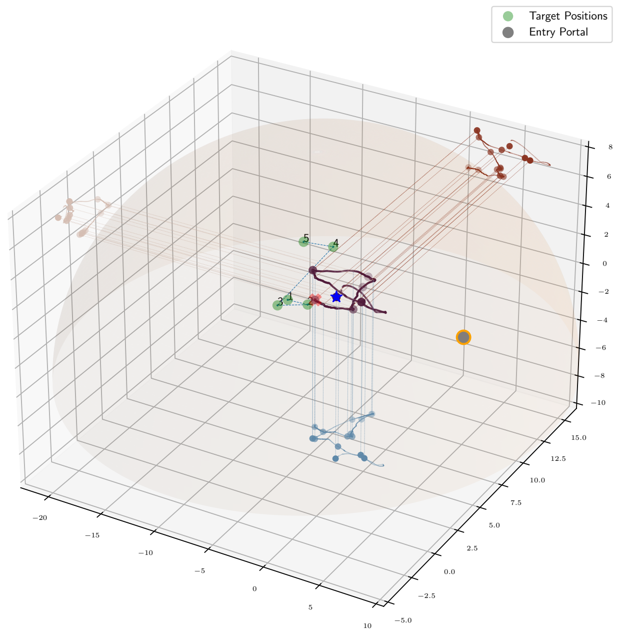

Fig. 15: Example expert trajectory for `Periscoping` task

Hyperparameter	`ImageCentering`	`Periscoping`	`TraceLines`
Batch size	256	1024	1024
Rollout buffer size	20480	20480	20480
Learning rate	5e-4	5e-4	5e-4
Entropy bonus β	0.01	0.01	0.01
Clipping threshold ϵ	0.2	0.3	0.3
GAE λ	0.95	0.95	0.95
Number of epochs	3	3	3
LR schedule	const	const	const
Network settings			
Normalise input	true	true	true
Number of hidden units	128	128	128
Number of layers	2	2	2
Reward signals			
Extrinsic γ	0.99	0.99	0.99
Extrinsic weight	1.0	1.0	1.0
Curiosity γ	0.99	0.99	0.99
Curiosity weight	0.15	0.2	0.1
Curiosity learning rate	3e-4	3e-4	3e-4
Curiosity hidden sise	128	128	256
IRL settings			
γ	0.99	0.99	0.99
Strength	1.0	1.0	1.0
Hidden sise γ	512	512	512
Learning rate	3e-4	3e-4	3e-4
Use actions	false	false	false

Table 10: PPO hyperparameters used for section 4 experiments

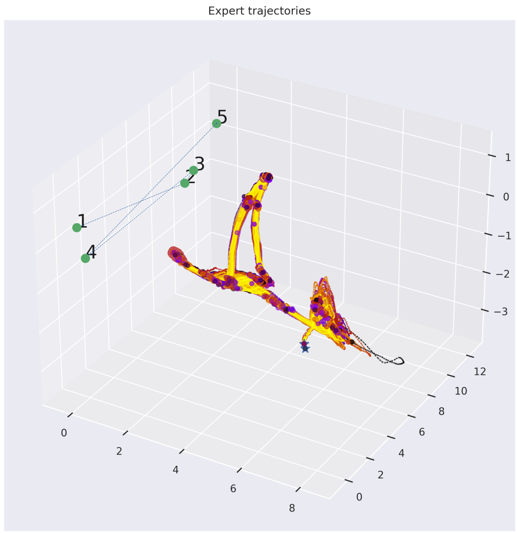

Fig. 16: 20 expert trajectories for `Periscoping` task

Hyperparameter	`ImageCentering`	`Periscoping`	`TraceLines`
Batch size	256	1024	1024
Replay buffer size	100000	100000	100000
Learning rate	3e-4	3e-4	3e-4
Initial temperature	0.2	1.0	1.0
τ	0.005	0.005	0.005
Steps per update ϵ	20	20	20
Reward signal steps per update ϵ	20	20	20
LR schedule	const	const	const
Network settings			
Normalise input	true	true	true
Number of hidden units	128	128	128
Number of layers	2	2	2
IRL settings			
γ	0.99	0.99	0.99
Strength	1.0	1.0	1.0
Hidden sise γ	256	256	256
Number of layers	2	2	2
Learning rate	3e-4	3e-4	3e-4
Use actions	false	false	false

Table 11: SAC hyperparameters used for section 4 experiments

Reward Features $\phi(s_i)$	w_i^{IC}	w_i^{PER}	w_i^{TL}
Progress bar active	0.2	2	0.1
Subtask completed	100	100	100
Exiting the dome	-1000	-1000	-1000
Velocity penalty	0.01	0.01	0.01
Distance penalty $\lvert\mathbf{x}_t^a - \mathbf{x}_t^t\rvert_2$	-0.01	-0.01	-0.01
Angle penalty $\lvert Q_t^a - Q_t^t\rvert_2$	-0.001	-0.001	-0.001
Target is visualised	0.01	0.01	0.01
Rays reward	-	0.001	-
Following spline	-	-	0.001

Table 12: Heuristic reward structure
